# Mechanistic Dissection of RNA-Binding Proteins in Regulated Gene Expression at Chromatin Levels

**DOI:** 10.1101/sqb.2019.84.039222

**Published:** 2020-01-03

**Authors:** Jia-Yu Chen, Do-Hwan Lim, Xiang-Dong Fu

**Affiliations:** Department of Cellular and Molecular Medicine, Institute of Genomic Medicine, University of California, San Diego, La Jolla, California 92093, USA

## Abstract

Eukaryotic genomes are known to prevalently transcribe diverse classes of RNAs, virtually all of which, including nascent RNAs from protein-coding genes, are now recognized to have regulatory functions in gene expression, suggesting that RNAs are both the products and the regulators of gene expression. Their functions must enlist specific RNA-binding proteins (RBPs) to execute their regulatory activities, and recent evidence suggests that nearly all biochemically defined chromatin regions in the human genome, whether defined for gene activation or silencing, have the involvement of specific RBPs. Interestingly, the boundary between RNA- and DNA-binding proteins is also melting, as many DNA-binding proteins traditionally studied in the context of transcription are able to bind RNAs, some of which may simultaneously bind both DNA and RNA to facilitate network interactions in three-dimensional (3D) genome. In this review, we focus on RBPs that function at chromatin levels, with particular emphasis on their mechanisms of action in regulated gene expression, which is intended to facilitate future functional and mechanistic dissection of chromatin-associated RBPs.

The traditional view of transcription is to produce either structural RNAs or protein-coding mRNAs. Specific structural RNAs are assembled into various RNA machines to catalyze specific biochemical reactions, and protein-coding RNAs are processed in the nucleus (such as capping, splicing, and polyadenylation) and then exported to the cytoplasm to translate into proteins. The advent of deepsequencing technologies has now revealed that mammalian genomes are far more active in transcription (Djebali et al. 2012), generating a large repertoire of regulatory RNAs, including long noncoding RNAs (lncRNAs) (Long et al. 2017), repeat-derived RNAs (Johnson and Straight 2017), and enhancer RNAs (eRNAs) (Li et al. 2016). Even protein-coding genes are producing various smaller RNA species that are either cause or consequence of regulated gene expression as a result of divergent or convergent transcription and transcription pausing and pause release (Wissink et al. 2019). Most regulatory RNAs are predominantly retained in the nucleus (Li and Fu 2019), where they may modulate gene expression at different steps of transcription on specific transcription units or genomic loci (Skalska et al. 2017), remodel chromatin structures and dynamics (Bohmdorfer and Wierzbicki 2015), and mediate long-distance genomic interactions (Schoenfelder and Fraser 2019), together contributing to the organization of the three-dimensional (3D) genome.

RNA molecules contain a series of single- and double-stranded regions that enable them to interact with DNA, RNA, and protein, thus providing versatile structural modules that are distinct from those in proteins to mediate network interactions. Through functional dissection of specific RNA metabolism pathways, a large number of RNA-binding proteins (RBPs) have been characterized, which often process unique structural motifs for direct contact with RNA sequences, base compositions and modifications, polynucleotide backbone, double-stranded regions, or RNA tertiary structures. However, recent global surveys of RBPs reveal ~ 1500 RBPs encoded by mammalian genomes, many of which do not carry canonical RNA-binding domains (Hentze et al. 2018). It is particularly interesting to note that many DNA-binding proteins are also able to directly bind RNA through either the same or distinct nucleic acid recognition motif(s), which are collectively termed DNA/RNA-binding proteins (DRBPs) (Hudson and Ortlund 2014). Consequently, many traditional DNA-binding transcription factors (TFs) may also function as RBPs in mammalian cells. These DRBPs are exemplified by many zinc-finger proteins, which often contain multiple fingers in the same polypeptides with divided tasks in interacting with DNA, RNA, and/or protein.

Given prevalent transcription activities in mammalian genomes, our recent large-scale chromatin immunoprecipitation sequencing (ChIP-seq) analysis of RBPs reveals that nearly all biochemically defined chromatin regions (based on RNA production, chromatin marks, and accessible chromatin regions) in the human genome involve specific RBPs, and a significant fraction of these nuclear RBPs appear to directly participate in transcriptional control (Xiao et al. 2019). In this review, we focus on RBPs that function at chromatin levels. We highlight recent advances in detecting RBP-chromatin interactions and in dissecting their mechanisms in transcriptional control and co-transcription RNA processing through acting on selective “hotspots” on chromatin to aid in future research to (i) understand a suspected function of an RBP on chromatin, (ii) probe the regulatory activity of a chromatin-associated RNA through identifying and characterizing its associated RBPs, (iii) dissect a specific chromatin activity that may involve both regulatory RNAs and RBPs, or (iv) deduce global DNA–RNA–protein networks in 3D genome critical for specific biological processes. Because of limited space, we select specific examples to illustrate how to experimentally approach the function and mechanism of chromatin-associated RBPs, rather than trying to be comprehensive in covering all related literature on regulatory RNAs and RBPs. Readers are directed to the outstanding reviews on such topics cited above.

## STRATEGIES TO DETECT CHROMATIN-ASSOCIATED RBPs

**Figure 1.**
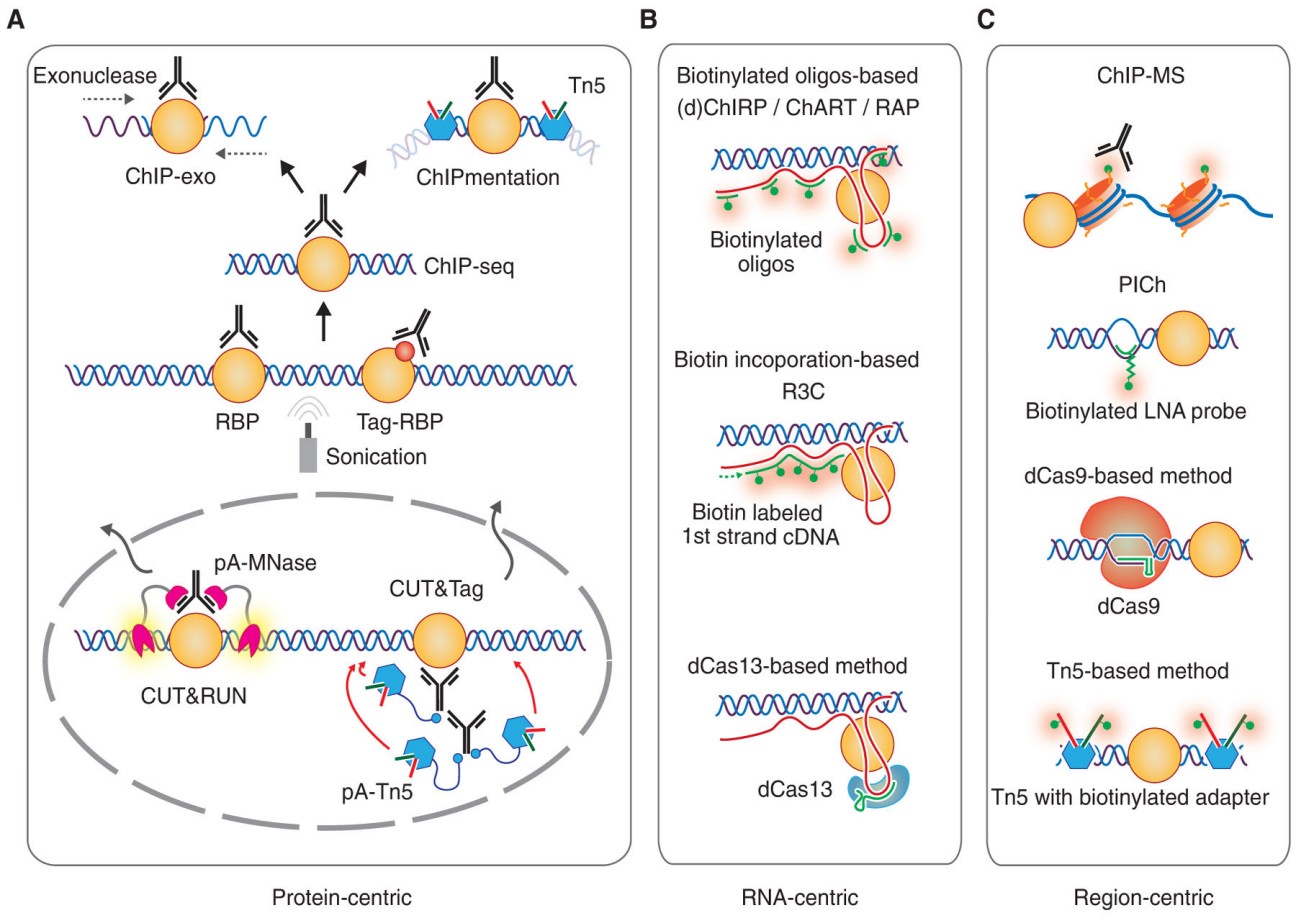
Strategies to detect chromatin-associated RNA-binding proteins (RBPs). (*A*) Protein-centric approaches to explore whether a specific RBP directly acts on chromatin. (*B*) RNA-centric approaches to identify both RBPs and associated DNA regions. (*C*) Regioncentric approaches to profile associated RBPs.

Chromatin-associated RBPs can be detected either on an individual basis or at the genome-wide scale. If the experimental goal is to explore a suspected function of a specific RBP on chromatin (Fig. 1A), the first step is to perform ChIP-seq if specific antibody is available or through genomic tagging using the CRISPR technology to determine the binding pattern of the RBP of interest on chromatin, essentially treating the RBP under investigation as a candidate TF. Options for genomic tagging include in-frame insertion of a GFP, FLAG, or SPY tag to the endogenous gene (Zakeri et al. 2012; Kimple et al. 2013). Multiple variations of ChIP-seq may be chosen, including ChIP-exonuclease (ChIP-exo) to increase the resolution (Rhee and Pugh 2011) or ChIPmentation to improve the robustness in library construction (Schmidl et al. 2015). More sophisticated variations include CUT&RUN and CUT&Tag, which use a Protein A-Micrococcal Nuclease or a Protein-A-Tn5 fusion protein to recognize chromatin-bound antibody (Skene and Henikoff 2017; Kaya-Okur et al. 2019). These techniques would avoid cross-linking and the harsh sonication step as in standard ChIP-seq. Deduced RBP-binding peaks can lead to functional and mechanistic studies through motif identification (Landt et al. 2012), Gene Ontology (GO)-term and Kyoto Encyclopedia of Genes and Genomes (KEGG) enrichment analyses (Kanehisa and Goto 2000; The Gene Ontology Consortium 2019), and cobinding analyses by using existing ChIP-seq data for known TFs and other RBPs (Xiao et al. 2019).

Before mechanistic dissection, two important questions may be addressed. The first is to determine whether the RBP of interest binds chromatin in an RNA-dependent manner. This can be addressed by using RNase A to treat permeabilized cells before ChIP-seq or using a drug, such as α-amanitin, to block transcription to determine the dependence on nascent RNA production. The second question is to identify specific RNAs that might mediate the interaction of the RBP with chromatin, which may not be as straightforward as it might sound. One approach is to sequence RNA, rather than DNA, in the IPed sample by RNA immunoprecipitation (RIP) (Gilbert and Svejstrup 2006) or formaldehyde RIP (fRIP) (Hendrickson et al. 2016). As these techniques do not differentiate direct from indirect binding, a better choice would be cross-linking immunoprecipitation (CLIP) (Lee and Ule 2018). Still, the problem is twofold: RBPs may bind RNAs both on and beyond chromatin and the RNA-binding profiles for most RBPs rarely match with those detected by ChIP-seq (Ji et al. 2013; Van Nostrand et al. 2018). One potential solution to this problem is to perform CLIP on biochemically enriched chromatin fractions, which may provide critical insights into RNA-guided interactions with DNA. This may be amenable with *cis*-acting RNAs, but it is quite challenging to link RBP–chromatin interactions mediated by *trans*-acting RNAs. However, this is readily approachable if a study begins with a specific chromatin-associated noncoding RNA and the goal is to understand the function and mechanism of such potential regulatory RNA on chromatin by identifying its interaction with DNA and then searching for potential RBPs involved (Fig. 1B). For this experimental goal, established RNA capture strategies could be used to identify the associated DNA and proteins, as exemplified by a set of related methods, such as ChIRP (Chu et al. 2011; Quinn et al. 2014), CHART (Simon et al. 2011), RAP (Engreitz et al. 2013), R3C (Zhang et al. 2014), and a potential dCas13-based approach similar to that using a dCas9-based strategy to target a specific genomic loci (Tsui et al. 2018). Additionally, RNA-linked chromatin architecture could be approached with HiChIRP (Mumbach et al. 2019), and RNA–RNA interactions with hiCLIP (Sugimoto et al. 2015). These technologies enable the elucidation of potential DNA-, RNA-, and protein-mediated network interactions in 3D genome.

Given that all active chromatin regions are suspected to involve regulatory RNAs and RBPs, it would require strategies to identify specific RNAs and RBPs at a specific genomic locus or genome-wide that are linked to a specific epigenetic event or a regulated gene expression program (Fig. 1C). A specific nucleic acid probe (Dejardin and Kingston 2009) or a dCas9-based strategy (Tsui et al. 2018) have been developed to detect locus-specific interactions by capture followed by genomic or proteomic profiling. To identify potential regulatory RNAs and RBPs associated with an epigenetic event, IP-coupled chromatin proteomic profiling would provide a general approach. This strategy has been applied to specific histone-marked genomic regions, uncovering multiple RBPs in complex with specific histone modification events (Ji et al. 2015). Conversely, regulated gene expression may enlist proteins that interact with nascent RNAs from various genomic regions, which can be approached by ethynyl uridine (EU) labeling to enable nascent RNAs to react with azide-biotin for streptavidin enrichment, which has uncovered many noncanonical RBPs that are well-known TFs and chromatin remodelers (Bao et al. 2018). In fact, EU may be combined with 4-thiouridine (4sU) labeling to enhance protein–RNA cross-linking (Huang et al. 2018). We may also envision a general strategy to systematically identify chromatin-associated RBPs by loading a biotinlabeled adaptor to Tn5 (Lai et al. 2018) to access all open chromatin regions followed by streptavidin enrichment and proteomic profiling. This approach would enable unbiased survey of annotated RBPs on chromatin in different cell types.

## DEFINING THE FUNCTIONAL IMPACT OF CHROMATIN-ASSOCIATED RBPs ON GENE EXPRESSION

Chromatin-associated RBPs may have direct roles in transcription or mediate cotranscriptional processing or both. It is thus critical to measure their functional impacts before investigating the mechanism of their actions. RNA-seq following knockdown has been typically used to quickly assess the functional consequence; however, the data do not necessarily reflect regulated gene expression at the level of transcription because steady state RNA is the collective consequence of transcription, RNA processing, and stability as well as indirect effects induced by knockdown of a specific RBP To determine potential impact on transcription, the most straightforward assay is global nuclear run-on (GRO-seq), which measures nascent RNA production (Core et al. 2008).

To help differentiate between direct versus indirect effects, one may determine whether RBP–chromatin interactions are linked to target genes the RBP binds, assuming that bound target genes are more affected than unbound genes. However, if the RBP under investigation preferentially binds intergenic regions, such as enhancers, it would be important to link individual binding events to likely target genes (Yao et al. 2015). Most studies infer the closest genes as targets for enhancers, which is reasonable for metagene analysis, but there are numerous exceptions to this assumption, as many enhancers may engage in long-distance interactions with target gene promoters through DNA looping, thus skipping the nearest neighboring genes (Schoenfelder and Fraser 2019). As transcription is a multistep process from the assembly of preinitiation complex (PIC) to transcription pausing and pause release to productive elongation to termination; various high-throughput technologies for analyzing different portions of nascent RNAs have been developed for mechanistic dissection. Readers are referred to a recent thorough review on these technologies and their applications to addressing specific mechanistic questions (Wissink et al. 2019).

If an RBP is suspected to play a direct role in transcription, it is often informative to survey its impact on the behavior of specific RNA polymerases. Using Pol II as an example, the carboxy-terminal domain (CTD) of the largest subunit is posttranslationally modified, and specific modification events have been linked to different steps in transcription according to the so-called CTD code (Hsin and Manley 2012). Therefore, quantifying those modification events and mapping them to chromatin by ChIP-seq in response to RBP knockdown are often informative to pinpoint a specific transcription step(s) being regulated (Takeuchi et al. 2018). Moreover, altered transcription is frequently linked to modified histones according to the histone code hypothesis (Jenuwein and Allis 2001), which can also be used to characterize the regulation of the epigenetic landscape by a specific chromatin-associated RBP.

Chromatin-bound RBPs are not necessarily involved in transcription, but rather in coupling transcription with downstream RNA processing events, which can affect RNA fates by multiple mechanisms. A battery of high-throughput technologies may be used to pinpoint changes in RNA fate. RNA-seq is again a powerful strategy to obtain the first approximation on differential gene expression. Resultant high-density reads can be aligned to the reference genome to deduce alternative splicing events by using rMATS (Shen et al. 2014). Various strategies to sequence the 3’ end of mRNAs can be used to quantify changes in steady state mRNAs as well as evaluate potential alternative polyadenylation (Zhou et al. 2014). Cotranscriptional RNA modifications can be determined by mapping specific modification events, such as m6A and ΨU, in mRNAs (Limbach and Paulines 2017). Functional impacts can be evaluated with 4sU-based methods in pulse-chase experiments for RNA stability (Tani and Akimitsu 2012), with subcellular fractionation and RNA sequencing (Frac-seq) for RNA export by performing separate RNA-seq analyses on cytoplasmic versus nuclear RNAs (Sterne-Weiler et al. 2013), and with ribosome profiling (Ribo-seq) for translational control (Ingolia et al. 2009). As an RBP may perform any of those functions independent of their association with chromatin, it has been a great challenge to determine whether their chromatin-binding activities contribute to specific functional impacts.

## ACTIONS AND MECHANISMS OF RBPs ON CHROMATIN

Below, we discuss specific RBPs that have been characterized to some mechanistic details to illustrate how RBPs may be coupled with regulatory RNAs to control gene expression via their actions on chromatin.

### RBPs as Part of RNA Polymerase Complexes

Multiple RBPs are known to be either part of the Pol II holoenzyme or PIC that contains Pol II. In fact, the active center cleft of Pol II has been found to be able to bind B2 noncoding RNA transcribed from a repeat (SINE) element (Kettenberger et al. 2006), which can potently inhibit transcription initiation (Espinoza et al. 2004). One of the Pol II subunit POLR2G (aka RPB7) contains a putative RNA-binding domain, which can bind DNA and RNA with similar affinity (Meka et al. 2005). As part of Pol II, this subunit sits close to the RNA exit channel and may play a critical role in transcription elongation. Interestingly, this Pol II subunit forms a heterodimer with POLR2D (aka RPB4), which has been recognized to modulate 3’-end formation of a subset of mRNAs in yeast, suggesting that it may play a critical role in polyadenylation-coupled transcription termination (Runner et al. 2008). By coIP, the RBP SFPQ has been reported to tightly associate with Pol II and modulate its phosphorylation in the Ser2 positions to influence transcription elongation (Takeuchi et al. 2018). Collectively, these findings illustrate that the Pol II complex has the capacity to bind RNA, either through its own active site or an RNA-binding subunit or a tightly associated RBP, thus rendering the Pol II machinery a direct target for modulation by regulatory RNAs.

### Promoters as Hotspots for RBP Actions

**Figure 2.**
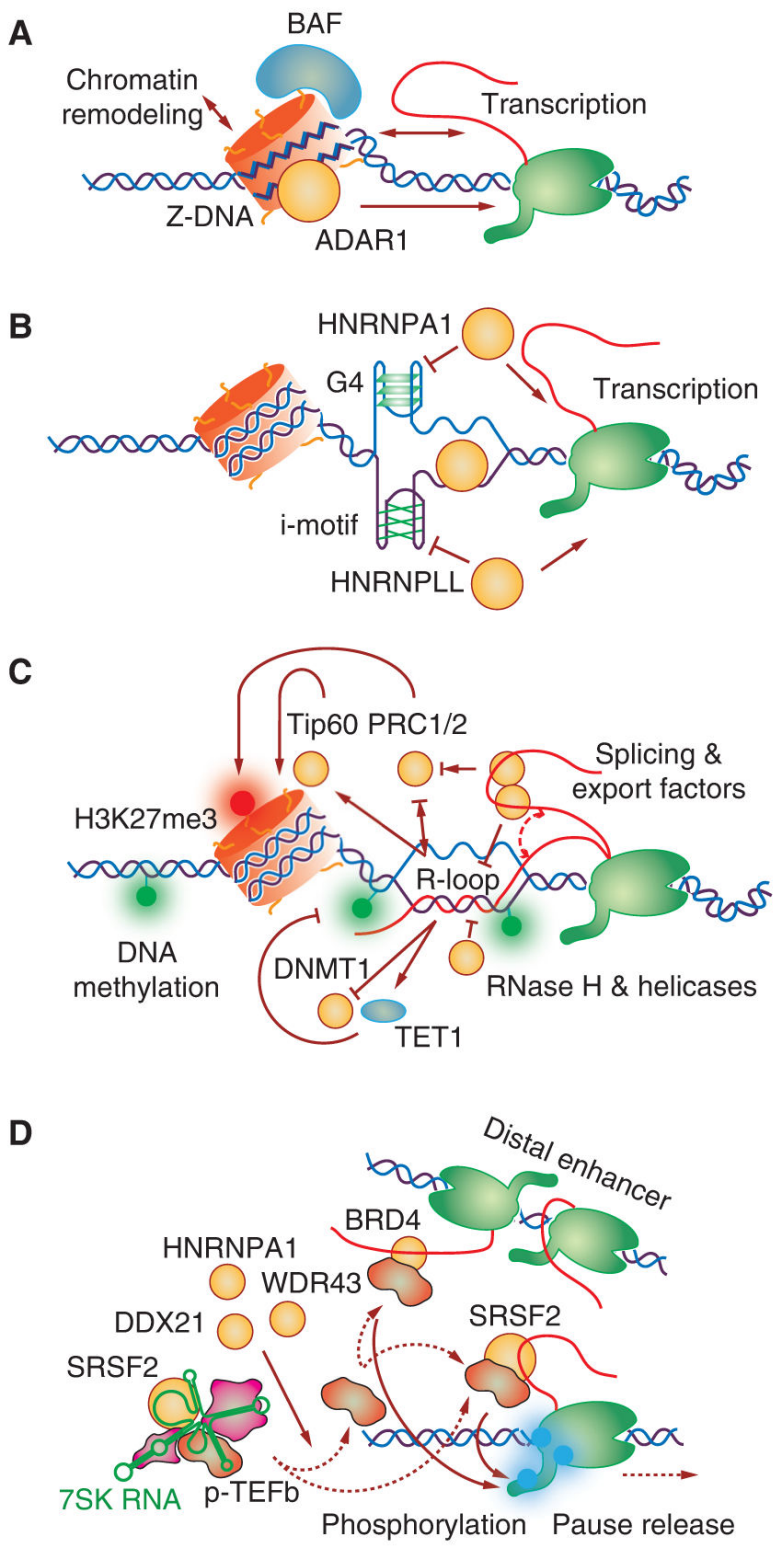
Mechanisms for RNA-binding proteins (RBPs) acting on promoters. (*A*) Mutual influence of Z-DNA formation and transcription, and regulation by the RNA editing enzyme ADAR1. (*B*) Recognition of G4, i-motif, or single-stranded DNA (ssDNA) by RBPs to regulate transcription. (*C*) The formation and resolution of R-loops, which can play positive roles in the recruitment of chromatin remodelers and DNA modification enzymes. (*D*) Multiple mechanisms for transcriptional pause release by relocating p-TEFb from 7SK complex to paused Pol II.

Initiation of transcription requires the accessibility of gene promoters to DNA-binding TFs, and a recent large-scale survey of RBPs reveals that gene promoters are also the most predominant hotspots for RBPs (Xiao et al. 2019). Although it is entirely conceivable that various downstream RNA processing events may begin via promoter-associated RBPs (see below), increasing evidence suggests that RBPs may have direct roles in facilitating chromatin accessibility to aid in transcription initiation. The formation of non-B DNA structure, such as Z-DNA, is known to contribute to the formation of nucleosome-free chromatin regions (Liu et al. 2006; Mulholland et al. 2012) to license transcription activation (Fig. 2A; Shin et al. 2016). Interestingly, ADAR1, a double-stranded RBP functioning in RNA editing (Nishikura 2010), appears to have the capacity to bind Z-DNA to enhance gene expression (Oh et al. 2002). Many gene promoters contain CpG islands. G-rich sequences have been suggested to form G-quadruplex (G4), and the RBP HNRNPA1 appears to help unfold such G4 DNA, thus altering the chromatin accessibility (Fig. 2B; Paramasivam et al. 2009). Interestingly, the opposite C-rich strand has also been postulated to form a fourstranded DNA referred to as “i-motif,” a structure that a recent study suggests does form in the cell (Zeraati et al. 2018), and HNRNPLL and several other RBPs appear to bind and unfold this motif to activate transcription (Fig. 2B; Kang et al. 2014; Abou Assi et al. 2018). The exposed single-stranded DNA (ssDNA) region at promoters is a potential platform for RBPs to act on, especially by those RBPs containing a KHdomain or RRM motif that has been long recognized to also bind ssDNA (Fig. 2B; Maris et al. 2005; Valverde et al. 2008). These findings together suggest that RBPs may participate in the formation and resolution of various non-B DNA structures to modulate chromatin accessibility, thus modulating transcription.

Most gene promoters in mammalian genomes are now known to be regulated by transcription pausing and pause release in promoter-proximal regions (Core and Adelman 2019). A large number of gene promoters contain CpG islands, which promote the formation of R-loop, a threestranded RNA/DNA structure in which nascent RNA anneals back to template DNA (Ginno et al. 2012). The ability of nascent RNA to invade into duplex DNA is greatly enhanced by the high propensity of nontemplate DNA to form G4-like structure, and, thus, R-loops are tightly associated with GC-skewed (G-rich sequence in nontemplate and C-rich sequence in template DNA) promoter regions (Chen et al. 2017b). R-loop formation is likely part of the mechanism for Pol II pausing (Chen et al. 2017b), which has been thought to repress gene expression and induce genome instability (Skourti-Stathaki and Proudfoot 2014). However, recent studies show that R-loop formation is also linked to gene activation, perhaps by facilitating the recruitment of chromatin remodeler, such as Tip60 (Fig. 2C; Chen et al. 2015), thereby enhancing TF binding (Boque-Sastre et al. 2015). Because RNA is a key participant in R-loop formation, various RBPs have been shown to modulate R-loop formation and/or resolution, which creates opportunities for RBPs to positively or negatively modulate transcription (Cristini et al. 2018; Wang et al. 2018). For example, the RNA helicases DDX21 and DDX5 have been suggested to help resolve R-loop to promote transcription (Fig. 2C; Argaud et al. 2019; Mersaoui et al. 2019). Pre-mRNA splicing and RNA export factors appear to help pull nascent RNAs out from R-loops to facilitate RNA processing and transport, thus preventing R-loop accumulation (Fig. 2C; Santos-Pereira and Aguilera 2015). Transient R-loop formation may also lead to sustainable changes in gene promoters by repulsing DNA methyltransferases DNMT1 (Grunseich et al. 2018) and recruiting DNA demethylase TET1 (Arab et al. 2019), together converting them to the hypomethylated state for gene activation (Fig. 2C). On the other hand, a recent study (Alecki et al. 2019) suggests that Polycomb complex 2 (PRC2) can help RNA invade into DNA to enhance R-loop formation and that Polycomb complex 1 (PRC1) can bind R-loop, together facilitating H3K27me3 deposition to silence gene expression (Fig. 2C).

The release of paused Pol II is a major step in transcriptional control, which is regulated by the P-TEFb complex, consisting of cyclin T and CDK9 kinase, to phosphorylate NELF, DSIF, and Pol II Ser2 (Saunders et al. 2006). Interestingly, P-TEFb is part of an inhibitory complex containing the 7SK noncoding RNA, which is associated with gene promoters, and releasing and relocating P-TEFb from the 7SK complex to the Pol II complex has been recognized to play a key role in Pol II pause release (Fig. 2D; Core and Adelman 2019). An increasing number of RBPs have been shown to be involved in this process. The RBP HNRNPA1 appears to promote the disassociation of P-TEFb from 7SK complex, thus tripping the kinase in promoter-proximal regions (Barrandon et al. 2007; Van Herreweghe et al. 2007). SR proteins, which have been extensively characterized as splicing commitment factors, are also part of the 7SK complex, which help extract P-TEFb from the 7SK complex and relocate this critical Pol II CTD kinase to nascent RNA to activate transcription (Ji et al. 2013). DDX21 has also been shown to release p-TEFb from the 7SK complex via its helicase activity (Calo et al. 2015), and more recently, another RBP WDR43 has been found to activate transcription by releasing P-TEFb from the 7SK complex (Bi et al. 2019). These findings suggest multiple mechanisms for releasing P-TEFb from the 7SK complex through coordinated actions of regulatory RNAs and RBPs. In fact, nascent RNA-induced P-TEFb release may also be part of the mechanism for eRNAs to activate transcription (Schröder et al. 2012; Chen et al. 2017a; Rahnamoun et al. 2018).

### RBPs to Facilitate Heterochromatin Formation, Spreading, and Maintenance

Gene silencing results from the formation of heterochromatin, but, counterintuitively, both the formation and maintenance of heterochromatin appear to depend on ongoing transcription. Heterochromatin can be further classified into facultative or constitutive heterochromatin, which are respectively decorated with H3K27me3 and H3K9me2/3. Interestingly, both lncRNAs and small RNAs have been shown to play critical roles in establishing heterochromatin (Li and Fu 2019). Exemplary analysis of X inactivation has provided critical insights into facultative heterochromatin formation and spreading, which is mediated by the lncRNA Xist (Wutz 2011). Through RNA pull-down coupled with mass spectrometric analysis, Xist has been shown to interact with multiple RBPs, particularly SPEN/SHARP, which appears to synergize with PRC2 in depositing H3K27me3 on targeted DNA regions (McHugh et al. 2015). Interestingly, all subunits of PRC2 have the capacity to directly bind RNA, thus contributing to both PRC2 recruitment and PRC2 spreading (Yan et al. 2019). PRC2 also interacts with nascent mRNAs; however, the functional consequence is still under active debate. The RBP RBFox2 has been shown to couple nascent RNA production with the recruitment of PRC2 as part of the feedback mechanism to maintain the bivalency for a subset of gene promoters (Wei et al. 2016), which are particularly prevalent in stem cells (Bernstein et al. 2006). The recently elucidated roles of Polycomb complexes in R-loop formation and recognition is consistent with RNA-guided H3K27me3 deposition in gene promoter regions (Alecki et al. 2019). On the other hand, when nascent RNAs are of sufficient abundance, they are able to evict PRC2 to prevent H3K27me3 deposition and thus help maintain genes in the highly active state (Kaneko et al. 2013; Beltran et al. 2016, 2019; Wang et al. 2017).

Repeat-derived small RNAs are well known to mediate the formation of constitutive heterochromatin (Matzke and Mosher 2014; Holoch and Moazed 2015; Johnson and Straight 2017). Briefly, in fission yeast, repeat-derived transcripts from active retrotransposons are amplified by RNA-dependent RNA polymerase and processed by Dicer to generate endo-siRNAs. In *Drosophila* germline cells, piRNAs are generated and amplified by the “ping-pong” mechanism. These endo-siRNAs or piRNAs are loaded on RNA-induced transcriptional silencing complex to target nascent homologous transcripts on chromatin, which help recruit H3K9me2/3 methyltransferases (SUV39) to deposit the histone marks to establish constitutive heterochromatin. Importantly, this process involves numerous RNA-mediated interactions, including direct interactions of the constitutive heterochromatin factors themselves with RNA to facilitate both the formation and spreading of heterochromatin (Muchardt et al. 2002; Johnson et al. 2017). It is thus conceivable that additional RBPs may be involved to fine-tune various steps, as illustrated by the role of the RBP Vigilin/HDLBP in binding hyperedited RNAs or other unstructured RNAs to enhance SUV39H1 recruitment (Zhou et al. 2008). Therefore, both RNAs and RBPs are instrumental to heterochromatin formation and maintenance, particularly in centromeric and pericentromeric regions, which is known to be critical for chromosome alignment during mitosis (Simon et al. 2015).

## CONNECTING TRANSCRIPTION TO DOWNSTREAM RNA METABOLISM EVENTS

The primary purpose for RBPs to associate with chromatin has been thought to facilitate cotranscriptional processing events from RNA modification to intron removal to polyadenylation to RNA export (Proudfoot et al. 2002; Bentley 2014). Although cotranscriptional RNA processing has been well-documented, the mechanisms are still poorly understood. A popular idea is that specific RBPs or RNA processing machineries may ride with elongating Pol II to facilitate cotranscriptional RNA processing. The CTD of the largest Pol II subunit is thought to play a key role in this process by providing a docking platform for various RNA processing machineries. However, whereas depletion of CTD did show profound impacts on capping, alternative splicing, and alternative polyadenylation (Fong and Bentley 2001), it remains to be determined whether the CTD is required for Pol II to interact with various RNA processing machineries. To our knowledge, this has only been documented with capping enzymes (McCracken et al. 1997).

Interestingly, promoters have been reported to dictate downstream events from splicing (Cramer et al. 1997; Moldón et al. 2008) to RNA stability (Bregman et al. 2011; Trcek et al. 2011) to RNA export (Xiao et al. 2019), and even translation in the cytoplasm (Zid and O’Shea 2014). These promoter-dependent RNA metabolic steps have been convincingly shown by promoter swap in budding yeast genome, but such strategy has not been applied to mammals. It is conceivable that specific promoter-associated RBPs may be switched to nascent RNAs according to the so-called recruitment model (Naftelberg et al. 2015), but to date, specific RBPs critical for such promoter-dependent RNA processing events have not yet been identified. Alternatively, different promoters may equip the Pol II machinery with different factors to influence elongation speed, thereby creating different windows of opportunities for positive and negative regulators to recognize emerging RNA signals to facilitate specific RNA processing events (Bentley 2014; Fong et al. 2014). This has been referred to as the kinetic model (Naftelberg et al. 2015), but the mechanism for this attractive model has remained poorly understood, which requires the identification of specific RNA processing regulators that recognize specific nascent RNA elements in a Pol II elongation speed-dependent manner.

Another popular idea is for RBPs to interact with various modified histones, thereby coupling specific epigenetic features to the regulation of RNA processing. Indeed, many RBPs have been identified to associate with histone modification events (Ji et al. 2015), and some potential H3K36me3 readers have been reported to mediate alternative splicing (Luco et al. 2010; Guo et al. 2014). However, evidence has remained relatively thin to support modified histones as a widespread coupling mechanism between transcription and cotranscriptional RNA processing, and, therefore, the epigenetic control of cotranscriptional RNA processing still largely remains as an attractive hypothesis. Interestingly, the converse scenario has also been documented by which cotranscriptional splicing appears also to influence specific histone modification events, such as H3K4me3 in gene promoters (Bieberstein et al. 2012) and H3K36me3 in gene bodies (de Almeida et al. 2011; Kim et al. 2011).

## ORGANIZING 3D GENOME FOR REGULATED GENE EXPRESSION PROGRAMS

**Figure 3.**
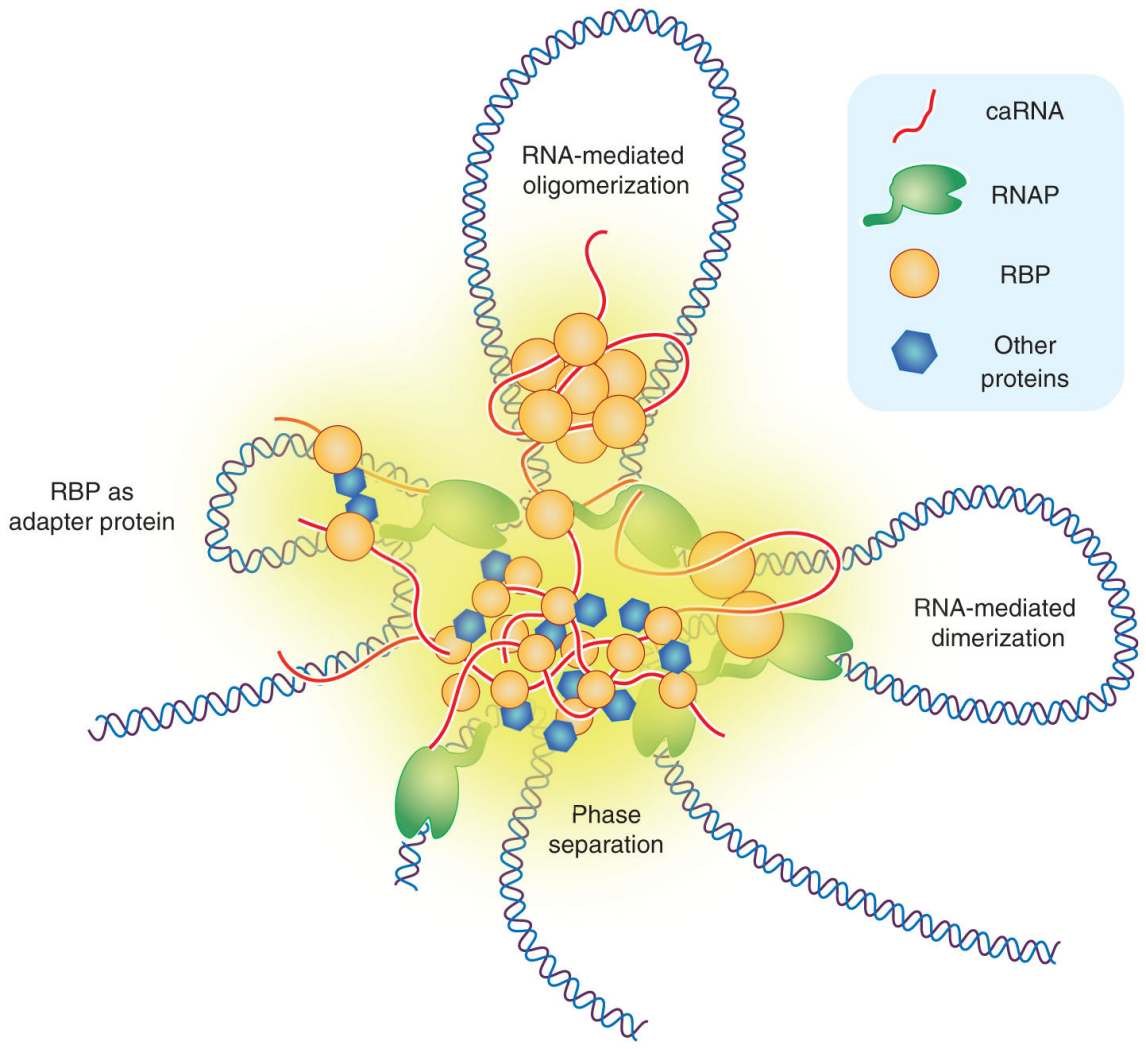
RNAs and RNA-binding proteins (RBPs) in 3D genome organization. Depicted are RNA-mediated dimerization (i.e., YY1), oligomerization (i.e., CTCF and HNRNPU), and genomic targeting (i.e., RBM25) for different TFs, together contributing to multivalent interactions to drive phase separation and formation of transcription hubs.

Chromatin-associated RBPs may play larger roles in 3D genome beyond their functions in modulating local transcriptional and cotranscriptional activities (Fig. 3), as regulatory RNAs have been increasingly recognized to provide multivalent interactions to coordinate chromosomal interactions (Li and Fu 2019). Chromosomes can be segregated into active A compartments and inactive B compartments (Lieberman-Aiden et al. 2009). Recent studies show that RNA-dependent oligomerization of the nuclear matrix–associated RBP HNRNPU plays key regulatory roles at the chromosome level (Nozawa et al. 2017; Fan et al. 2018). Deletion of HNRNPU weakens the boundary between A and B compartments, leading to A-to-B switches and overall chromosome condensation (Nozawa et al. 2017; Fan et al. 2018). HNRNPU appears to work with various chromatin-associated RNAs to help organize 3D genome, including some lncRNAs, such as FIRRE (Hacisuleyman et al. 2014), Xist (Hasegawa et al. 2010), and double-stranded viral RNAs (Cao et al. 2019). As 3D genome involves numerous long-range interactions, chromatin-associated RNAs and RBPs may bridge such interactions (Kim and Shendure 2019). CTCF, one of the best-known high-order chromatin organizers, is able to bind DNA and RNA (Sun et al. 2013; Saldaña-Meyer et al. 2014), which has recently been shown to be mediated by distinct zinc fingers (Hansen et al. 2019; Saldaña-Meyer et al. 2019). As zinc fingers are also involved in protein–protein interactions, CTCF appears to form oligomers through the same zinc finger for RNA binding, and disruption of this domain greatly impairs specific long-range genomic interactions (Hansen et al. 2019; Saldaña-Meyer et al. 2019), suggesting that CTCF may mediate critical RNA–protein–DNA interaction networks in 3D genome.

More recently, another zinc-finger-containing TF YY1 is recognized to also play a broad role in mediating long-distance genomic interactions between promoters and enhancers (Weintraub et al. 2017). Interestingly, YY1 is also able to bind RNA, which appears to be required for its efficient targeting to specific promoters and enhancers (Sigova et al. 2015). A recent large-scale cobinding analysis between TFs and RBPs on chromatin reveals that YY1 colocalizes with the RBP RBM25 on chromatin in which RBM25 appears to direct YY1 to target genomic loci (Xiao et al. 2019). Thus, unlike CTCF, which can bind both DNA and RNA, the genome organization function of YY1 is mediated through its partnership with a specific RBP. It is attempting to speculate that various TFs may functionally interact with specific RBPs to increase the specificity and/or efficiency in genomic targeting, which is in line with the emerging concept for the formation of transcription hubs that result from multivalent interactions to induce liquid–liquid phase transition for gene activation in the nucleus (Hnisz et al. 2017; Boija et al. 2018; Sabari et al. 2018; Cramer 2019). RBPs may play critical roles in this process (Saha et al. 2016; Maharana et al. 2018), as RBPs are highly enriched with intrinsically disordered regions (Castello et al. 2012), which have been shown to be key driving forces for phase separation (Molliex et al. 2015; Lin et al. 2017). Therefore, 3D genome is likely orchestrated by network interactions among DNA, RNA, TFs, and RBPs to drive cell type–specific gene expression programs (Guo et al. 2019).

## CONCLUSION

We have particularly focused in this review on diverse functions and mechanisms of chromatin-associated RBPs in regulated gene expression. An important message is that RNAs are no longer just products, but are also regulators of gene expression. Their regulatory functions are executed by specific RBPs, together contributing to network interactions in 3D genome. Such RNA and RBP-mediated interactions are critical for both gene activation and silencing, which calls for future research in this direction to understand how functional genome is organized and dynamically regulated by RNAs and RBPs in development and disease.
